# VALIDITY AND RELIABILITY OF THE MULTIDIMENSIONAL FUNCTIONAL ASSESSMENT QUESTIONNAIRE (OARS) IN ANGOLAN ELDERLY

**DOI:** 10.1093/geroni/igae098.3926

**Published:** 2024-12-31

**Authors:** Rita Bárbara, Rui Gonçalves, Carlos Sousa, Luciano Chingui, Antonio Armando, Mario Lopes

**Affiliations:** Universidade de Aveiro, Aveiro, Portugal; Escola Superior de Tecnologia da Saúde de Coimbra do Instituto Politécnico de Coimbra., Coimbra, Portugal; Universidade Privada de Angola, Luanda, Angola; Universidade Metodista Angola, Luanda, Angola; Universidade Privada de Angola, Luanda, Angola; Universidade de Aveiro, Aveiro, Portugal

## Abstract

**Introduction:**

The Older American Resources and Services (OARS) questionnaire assesses functionality and cognition in the elderly. Validating and culturally adapting instruments like the OARS is crucial to ensure their accuracy across different contexts. Objective: This study aimed to translate, culturally adapt, and validate the OARS for elderly individuals in Angola, focusing on cognitive and functional domains.

**Methods:**

The adaptation of the OARS involved a rigorous process, including translation, cultural adaptation, and clinimetric analysis. A multidisciplinary panel of experts, including professionals from internal medicine, public health, epidemiology, physiotherapy, and nursing, reviewed and validated the adapted version. The sample comprised 102 elderly residents of Luanda, randomly selected to ensure representativeness. Reliability was assessed using Cronbach’s alpha coefficient, while validity was examined through exploratory factor analysis, with particular attention to item factor loadings.

**Results:**

The physical activity domain demonstrated high internal consistency (Cronbach’s alpha = 0.80) and robust factor loadings, indicating reliability and validity in the Angolan context. The mental health domain showed moderate internal consistency (Cronbach’s alpha = 0.59) and variable factor loadings, suggesting the need for further refinement for better cultural and linguistic adaptation.

**Conclusion:**

The adapted OARS version proved reliable for assessing physical activity among Angolan elderly, but the mental health domain requires adjustments. These findings highlight the importance of clinimetrics and factor analysis in the cultural adaptation of instruments, ensuring their effectiveness across diverse sociocultural contexts. Keywords: Cognition, clinimetrics, validation

